# Physiological and Pathophysiological Responses to Ultramarathon Running in Non-elite Runners

**DOI:** 10.3389/fphys.2019.01300

**Published:** 2019-10-17

**Authors:** Florian Hoppel, Elisa Calabria, Dominik Pesta, Wilhelm Kantner-Rumplmair, Erich Gnaiger, Martin Burtscher

**Affiliations:** ^1^Oroboros Instruments, Innsbruck, Austria; ^2^Department of Sport Science, University of Innsbruck, Innsbruck, Austria; ^3^Department of Neurosciences, Biomedicine and Movement Sciences, University of Verona, Verona, Italy; ^4^Institute for Clinical Diabetology, German Diabetes Center, Leibniz Institute for Diabetes Research at Heinrich-Heine University, Düsseldorf, Germany; ^5^German Center for Diabetes Research, München-Neuherberg, Germany; ^6^Psychosomatic Pain Ambulance, University Hospital for Medical Psychology and Psychotherapy, Innsbruck, Austria; ^7^D. Swarovski Research Laboratory, Department of Visceral, Transplant Thoracic Surgery, Medical University Innsbruck, Innsbruck, Austria

**Keywords:** ultramarathon running, health effects, immune system, acute kidney injury, muscle damage

## Abstract

Ultramarathon running represents a major physical challenge even for elite athletes. Runners wellbeing may be challenged by fluid and electrolyte disturbances, hemolysis and skeletal muscle damage, decline in hepatic function and kidney injury. We hypothesized that these effects may even be exacerbated in non-elite runners. Physiological, hematological and biochemical parameters of ten males (26–45 years, weekly training time 8.5 h), participating in a mountain ultramarathon (67 km; approximately 4,500 m of total ascent), were determined before (PRE), immediately after finishing the ultramarathon (POST), and 24 h after the individual finish (REC). Race times of the 8 finishers (2 drop-outs due to hot ambient temperature) varied between 10.4 and 16.1 h, which almost represents the range of the entire starter field (8.82 h–17.47 h). The following changes in mean values of selected markers for skeletal muscle damage and kidney injury were observed from PRE to POST: creatine kinase (CK) + 1289%, lactate dehydrogenase (LDH) + 87%, serum creatinine (CR) + 72%, blood urea nitrogen (BUN) + 96%, and estimated glomerular filtration rate (eGFR) – 45%. Values of CK + 1447%, LDH + 56%, and BUN + 71% remained elevated at REC. White blood cells were increased (+ 137%) only POST. In conclusion, CK and LDH levels and leucocytosis may be considered to be relatively harmless “side-effects” of prolonged running in this group of male subjects with rather moderate ultramarathon experience and training status. However, acute kidney injury may become clinically relevant in this population under the certain conditions, which should be considered by responsible race managers and medical advisors.

## Introduction

Long-distance endurance competitions, particularly ultramarathon runs, are challenging events, potentially affecting various physiological functions (Knechtle and Nikolaidis, 2018). Many studies have analyzed physiological and pathophysiological responses to rather moderate running distances such as half-marathons (21.1 km) or traditional marathons (42.2 km) (Tian et al., 2011; Traiperm et al., 2013; Shin et al., 2016). In contrast, ultramarathons (distances > 42.2 km including a high variety of different course profiles) have been rarely studied. However, they represent an outstanding opportunity to evaluate adaptive responses to extreme loads and stress (Millet and Millet, 2010; Eichenberger et al., 2012). Differences in distance covered and running intensity of various competitions (half-marathons, marathons, and ultramarathons) may differently affect physiological responses and trigger different pathological events in adult non-elite runners (Jastrzębski et al., 2016; Shin et al., 2016).

For instance, fluid and electrolyte disturbances, hemolysis and skeletal muscle damage can occur during long-distance running (Knechtle and Nikolaidis, 2018). Body mass (BM) reduction due to fluid loss is a common observation after ultramarathon running (Kao et al., 2008; Knechtle and Nikolaidis, 2018). A slight reduction (≤ 2%) is usually not associated with impaired running performance and/or significant fluid and electrolyte disturbances (Cheuvront et al., 2007; Hou et al., 2015). In contrast, BM loss in excess of 2% may negatively affect exercise performance (Casa et al., 2005; Cheuvront et al., 2007; Sawka et al., 2007; Stearns et al., 2009). Loss of BM is determined by the sweating rate and fluid consumption during the competition but can also be caused by loss of solid body mass (skeletal muscle mass, fat mass) (Knechtle et al., 2012). The amount of fluid loss due to sweating in marathon running is highly variable and can exceed 2.8 L/h (Costill, 1977). The average loss of sweat during marathon running was reported to range from 0.83 L/h (< 15°C ambient temperature) to 1.2 L/h (warm weather, > 15°C), primarily depending on the duration and intensity of exercise and the ambient temperature (Cheuvront and Haymes, 2001). Sodium sweat concentration is variable as well and is determined by sweat rate, heat acclimation and hydration, averaging ∼50 mmol/L (range: 10–100 mmol/L) (Allan and Wilson, 1971; Sawka et al., 2007). Compared to blood reference range (136–145 mmol/L) sweat is hypotonic (Kratz et al., 2004), but can trigger electrolyte disturbances when sweat loss cannot be compensated by adequate fluid ingestion. Thus, not replacing sweat-induced fluid loss will cause hypernatremia and drinking solely water will provoke hyponatremia, which is frequently observed in ultramarathon running (Meinders and Meinders, 2007; Sawka et al., 2007; Hoffman et al., 2012; Krabak et al., 2014, 2017). Both types of electrolyte disturbances may have either relatively harmless or even severe health consequences such as headache, nausea, and vomiting, or even seizures, cerebral and pulmonary edema or death (Ayus et al., 2000; Hsieh et al., 2002; Krabak et al., 2014, 2017).

Hemolysis due to repetitive foot strikes and/or gastrointestinal bleeding may cause runner’s anemia (Kratz et al., 2006; Ottomano and Franchini, 2012; Traiperm et al., 2013) and microtrauma in skeletal muscle may even trigger rhabdomyolysis, indicated by increases in plasma creatine kinase (CK) and lactate dehydrogenase (LDH) levels after marathon running (Kobayashi et al., 2005; Tian et al., 2011). Muscle damage induces systemic inflammation (e.g., indicated by elevated IL-6 levels) and activates the immune system, e.g., indicated by elevated white blood cell count (leucocytosis) (Suzuki et al., 2003; Kasprowicz et al., 2013; Traiperm et al., 2013). Markers of impaired kidney function or kidney injury [e.g., creatinine (CR), blood urea nitrogen (BUN), glomerular filtration rate (GFR)] support the occurrence of rhabdomyolysis to clinical relevant levels in up to 40% of marathon finishers (McCullough et al., 2011; Shin et al., 2016; Traiperm et al., 2016).

How ultramarathon running can affect these parameters, especially in non-elite runners, has been less investigated. The severity of skeletal muscle damage, liver and kidney function, seem to be directly related to the distance covered, e.g., 42.2 vs. 100 vs. 308 km (Wu et al., 2004; Kim et al., 2007; Shin et al., 2016). However, severe muscular damage has not been demonstrated in every study (Lippi et al., 2012; Bird et al., 2014; Shin et al., 2016; Belli et al., 2018). Also, sports anemia and hyponatremia seem to occur slightly more frequently after completing ultramarathons compared to shorter distances (Knechtle et al., 2011; Lippi et al., 2012; Scotney and Reid, 2015; Cairns and Hew-Butler, 2016; Jastrzębski et al., 2016). Despite time of workload as widely accepted parameter affecting levels of serum enzymes (primarily known for muscle and liver damage markers), it has become evident that age, total muscle mass, climate conditions and type of workload (eccentric workload as during downhill running) and level of experience are also key factors in determining systemic changes to acute stress induced by prolonged running (Kim et al., 2009; Malm et al., 2004; Hou et al., 2015; Jastrzębski et al., 2015, 2016). For instance, higher age, increased exercise intensity and eccentric workload were shown to aggravate liver and muscle damage after ultramarathon running, whereas white blood cells and serum electrolyte concentration and metabolic consequences were found to change similarly in all ages and training groups (Jastrzębski et al., 2015, 2016).

The individual systemic changes following ultramarathon running are dependent on various parameters. However, only very few data are available on physiological responses and/or adverse health effects in adult non-elite ultramarathon runners. Thus, the aim of the present study was to investigate physiological and pathophysiological responses in adult non-elite ultramarathon participants. We hypothesized that a large proportion of these individuals would report clinically relevant levels of markers of skeletal muscle damage, inflammation, fluid and electrolyte disturbances and impairment of kidney function.

## Materials and Methods

### Study Participants

All local runners in the age group from 20 to 50 were selected from the entry list of male starters. Since measurements were performed on 3 consecutive days, it was only feasible for locals to participate in the present study. The 32 starters available were contacted 4 weeks prior to the event. They were informed about the procedure of measurements and asked to fill a questionnaire on anthropometric data, training and medical history, and smoking behavior. Exclusion criteria were any disease associated with an increased risk due to the ultramarathon running event (assessed by an experienced physician), and very high training volumes (a recreational athlete was here defined as a person training < 15 h/week) with the intention to recruit only recreational participants. Ten athletes (training volume: 4–15 h, median: 8.5 h) fulfilled all inclusion criteria and were included after providing written informed consent. The study was approved by the local ethics review board (University of Innsbruck, Institute of Sport Science).

### Competition Profile and Determination of Race Time

**FIGURE 1:**
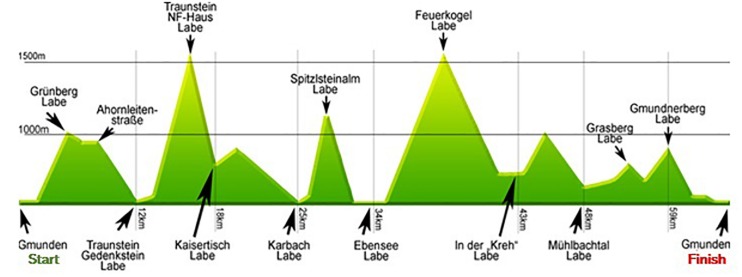
Race profile and refreshment stations throughout the course. Labe, refreshment station during the course.

The competition was performed as a non-stop mountain ultramarathon (67 km distance and approximately 4,500 m of total ascent and descent, and the highest point of the course was 1590 m a.s.l), taking place annually in Gmunden, Upper Austria (Figure 1). Race participants could choose their starting time individually between 3:00 and 5:00 am; cut off for finishing the race was set at 9:00 pm at the same day. Food and beverages (water, soft drinks and sports drinks, soups, fruits, sweets, energy bars) were provided by the organizers at specific stations approximately every 5 km along the race course. 210 starters were men and only 19 women, and the drop-out rate was 26% in men and 21% in women.

Participants were wearing a heart rate monitor during competition with a recording interval of one second (Suunto Ambit 3; Suunto, Vantaa, Finland) to monitor race times, and average and maximal heart rates during the race (Suunto Movescount). Race time and average running speed were calculated using exact start and finishing times. We were not able to monitor food and fluid intake throughout the competition due to the wide spread of refreshment stations throughout the course. Competitors were asked POST to estimate their liquid intake.

**FIGURE 2:**
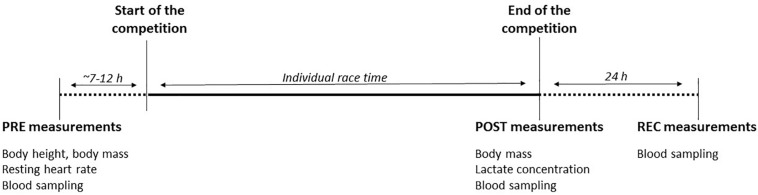
Timeline of measurements. PRE, baseline measurements performed ∼7–12 h before race start; POST, post-race measurements performed within 10 min after finishing; REC, recovery measurements performed 24 h after individual finishing. Recorded parameters are given for each measurement.

Blood sampling was performed by the same physician on the day before the competition (PRE), within 10 min after finishing the race (POST) and 24 h after the individual finish (REC). Body height and resting heart rate measurements were performed PRE, body mass was determined PRE and POST. Blood lactate concentrations were only determined POST. See Figure 2 for the timeline for all measurements.

### Blood Analyses: Red and White Blood Cell Count, Electrolyte Concentration and Markers for Muscle and Kidney Injury

Whole blood was taken from the median cubital vein using different blood collection tubes (∼9 mL K3 EDTA, 3 mL Lithium Heparin, 3 mL clot activator for serum analysis; BD Vacutainer, BD diagnostics, New Jersey, United States). The tubes were stored at 4°C and transported to the local hospital for analysis on each sampling day (Salzkammergut-Klinikum, Gmunden, Austria). Red and white blood cell counts were determined by automated counting (Siemens Advia 120, Siemens, Vienna, Austria). Blood electrolyte concentration [potassium (K), sodium (Na), chloride (Cl), calcium (Ca), magnesium (Mg)], blood glucose levels (BG), indirect markers of muscular damage (CK, LDH) and parameters of renal and liver function (CR, BUN) were assessed by photometry and V-Lyte technology (Dimension Vista 500, Siemens, Vienna, Austria). GFR was calculated from CR through CKD-EPI formula (chronic kidney disease epidemiology collaboration), considering race, sex and age (Stevens and Levey, 2005; Levey et al., 2009).

The severity of acute kidney injury was classified according to AKIN criteria published by Acute Kidney Injury Network: Stage I (consistent with an increase in CR PRE-POST of minimum 0.3 mg/dL or 1.5 to twofold from pre-race level), stage II (increase in post-race CR 2 to threefold from PRE) and stage III (increase of > threefold or minimum 4 mg/dL CR with an acute increase in CR of 0.5 mg/dL) (Mehta et al., 2007). All reference ranges used are presented in Table 1 and are standard references with respect to sex and ethnic group in white blood cells (WBC) and platelets (PLT), and to athletic performance in CK (Brain, 1996; Kratz et al., 2004; Mougios, 2007).

**TABLE 1:** Reference ranges for blood parameters.

**Blood cell counts and hematocrit**		**Blood electrolytes**		**Parameters to indicate muscular**	
				**damage and kidney function**	
RBC [10^6^ μL^–1^]	4.5–5.9	Na [mmol⋅L^–1^]	136–145	CK [U/L]	60–400/<1083
WBC [10^3^ μL^–1^]	3.6–9.2	Cl [mmol⋅L^–1^]	98–106	LDH [U/L]	100–190
NEU [10^3^ μL^–1^]	1.7–6.1	K [mmol⋅L^–1^]	3.5–5.0	CR [mg/dL]	<1.5
MONO [10^3^ μL^–1^]	0.18–0.62	Ca [mmol⋅L^–1^]	2.1–2.7	BUN [mg/dL]	10–20
LYM [10^3^ μL^–^]	1.0–2.9	Mg [mmol⋅L^–1^]	0.7–1.0	GFR [mL/min/1.73 m^2^]	>90
PLT [10^3^ μL^–^]	143–332			BG [mg/dL]	<126 mg/dL
Hct [%]	41.0–53.0				

*RBC, red blood cells; WBC, white blood cells; PLT, platelets; NEU, neutrophils; LYM, lymphocytes; MONO, monocytes; Hct, hematocrit; K, potassium; Na, sodium; Cl, chloride; Ca, calcium; Mg, magnesium; CK, creatine kinase; LDH, lactate dehydrogenase; CR, serum creatinine; BUN, blood urea nitrogen; GFR, glomerular filtration rate; BG, blood glucose. Creatine kinase: 60–400 U/L is the clinical standard reference, < 1083 U/L is for active athletes (Mougios, 2007).*

### Statistics

**FIGURE 3:**
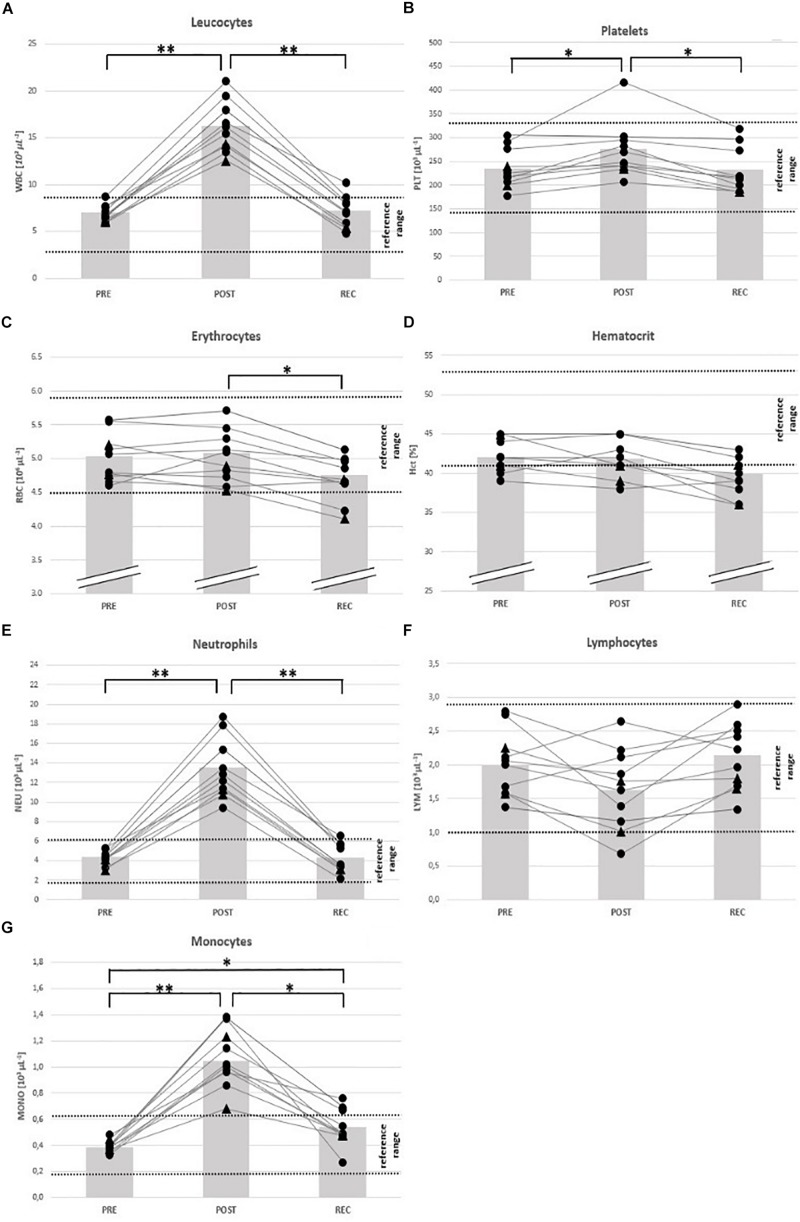
Individual data on blood cell counts and hematocrit before competition (PRE), immediately after the race (POST) and 24 h after finishing (REC). Changes of leucocytes **(A)**, platelets **(B)**, erythrocytes **(C)**, hematocrit **(D)**, neutrophils **(E)**, lymphocytes **(F)**, and monocytes **(G)**. Drop-outs marked triangular. ^∗^*p* < 0.05, ^∗∗^*p* < 0.01.

Athletes anthropometric data (Table 2) and race performance (Table 3) are presented as median and interquartile range (IQR), being the most robust in outliers. Age and race performance of study participants are given in comparison to all male finishers of the competition. Results of all blood parameters are shown in Table 4 as mean ± standard deviation (*SD*). Minimum, maximum and number of participants out of reference range are given in Table 5 for each parameter. Individual data are shown in Figure 3 (blood cell count, hematocrit), 4 (blood electrolytes and blood glucose) and 5 (muscular damage and renal function). To analyse changes from PRE to POST and POST to REC repeated one-way analysis of variance (ANOVA) with *post hoc* analysis (Bonferroni correction) was used for normally distributed data. Wilcoxon signed-rank test and Friedman test were applied for data not meeting ANOVA test requirements. Percentage differences between measurements (PRE-POST, POST-REC, PRE-REC) were calculated to present changes. All values were checked for clinical relevance (out of reference, OOR; used symbols: above upper range limit, (↑); below lower range limit, (↓); see Table 1 for reference ranges). Due to the hot weather conditions, two participants did not complete the race and thus 8 individuals were included in the final analysis, yet POST and REC values were measured in the 2 drop-outs. These data are not included in the statistics but are shown in the graphs using different symbols. Statistics were performed using IBM SPSS Statistics 24.0 (SPSS Inc., Chicago, IL, United States). A *p*-value below 0.05 was considered statistically significant.

**TABLE 2:** Anthropometric characteristics of participants.

		***N* = 8**			
		
		**Median**	**Min**	**Max**	**IQR**
Age [years]		41.5	26	45	13.5
Height [cm]		180	173	191	11
Body mass [kg]	PRE	74.5	67.0	90.0	15.3
	POST	74.4	66.9	87.6	14.4
BMI [kg/m^2^]	PRE	24.0	20.7	28.1	4.2
	POST	23.8	20.7	28.1	4.0
Resting heart rate [bpm]		68	38	75	20.8
Training/week [h]		8.5	4	15	5.3

*Data are presented as median, minimum (Min), maximum (Max), interquartile range (IQR). Drop-outs not included.*

**TABLE 3:** Age and race performance of study participants compared to all male finishers.

	**Study participants (*N* = 8)**				**Race participants (*N* = 162)**			
		
	**Median**	**Min**	**Max**	**IQR**	**Median**	**Min**	**Max**	**IQR**
Age [yrs]	41.5	26	45	13.5	42.0	17	75	17.0
Race time [h]	13.33	10.37	16.05	2.92	13.58	8.82	17.47	3.55
Speed [km/h]	5.1	4.2	6.5	1.2	5.0	3.6	7.8	1.5
HR min [bpm]	95	67	110	35.5				
HR avg. [bpm]	138	123	145	17.0				
HR peak [bpm]	177	160	183	16.0				
Lactate [mmol/L]	2.6	2.0	4.8	1.6				

*Data are presented as median, minimum (min), maximum (max), interquartile range (IQR). HR min, minimal heart rate; HR avg, average heart rate; HR peak, maximal heart rate, all during competition; lactate, post-race lactate. 2 of 10 study participants (20%) and 55 of 210 race participants (26%) did not finish the race; drop-outs not included.*

**TABLE 4:** Blood cell counts, serum electrolytes, tissue damage and renal function PRE, POST and REC.

			**After**				
				
	**PRE**	**95% CI**	**POST**	**95% CI**	**REC**	**95% CI**	***p***
**Blood cell count, hematocrit**							
Erythrocytes [10^6^ μL^–1^]	5.02 ± 0.38	[4.75–5.28]	5.10 ± 0.38	[4.84–5.37]	4.76 ± 0.28	[4.57–4.96]	0.002^×^
Leucocytes [10^3^ μL^–1^]	7.07 ± 0.89	[6.45–7.68]	16.75 ± 2.60^∗∗^	[14.95–18.56]	7.43 ± 1.66	[6.28–8.58]	< 0.001^×^
Neutrophils [10^3^ μL^–1^]	4.42 ± 0.66	[3.96–4.87]	13.90 ± 3.20^∗∗^	[11.68–16.11]	4.46 ± 1.52	[3.41–5.51]	< 0.001^×^
Monocytes [10^3^ μL^–1^]	0.39 ± 0.04	[0.36–0.42]	1.09 ± 0.19^∗∗^	[0.95–1.22]	0.6 ± 0.16**†**	[0.44–0.66]	< 0.001^×^
Eosinophils/Basophils [10^3^ μL^–1^]	0.21 ± 0.12	[0.14–0.30]	0.07 ± 0.09	[0.01–0.11]	0.26 ± 0.19	[0.08–0.35]	0.034^×^
Lymphocytes [10^3^ μL^–1^]	2.04 ± 0.52	[1.68–2.40]	1.71 ± 0.63	[1.27–2.15]	2.21 ± 0.51	[1.85–2.56]	0.057
Platelets [10^3^ μL^–1^]	239.1 ± 45.63	[207.50–270.75]	282.1 ± 62.57^∗∗^	[238.76–325.49]	239.9 ± 48.98	[205.93–273.82]	0.008^×^
Hematocrit [%]	41.8 ± 1.98	[40.38–43.12]	42.0 ± 2.33	[40.39–43.61]	39.8 ± 2.25	[38.19–41.31]	0.007^×^
**Blood electrolytes, blood glucose**							
Sodium [mEq⋅L^–1^]	140.13 ± 1.64	[138.99–141.26]	137.50 ± 5.42	[133.74–141.26]	139.50 ± 2.83	[137.54–141.46]	0.269
Chloride [mEq⋅L^–1^]	100.38 ± 1.69	[99.21–101.54]	95.63 ± 5.73	[91.65–99.60]	100.25 ± 3.2	[98.04–102.46]	0.027^×^
Potassium [mEq⋅L^–1^]	4.48 ± 0.47	[4.15–4.80]	4.89 ± 0.62	[4.45–5.32]	5.04 ± 0.71	[4.55–5.53]	0.014^×^
Calcium [mmol/L]	2.49 ± 0.10	[2.42–2.56]	2.80 ± 0.10^∗∗^	[2.73–2.87]	2.40 ± 0.07**†**	[2.35–2.45]	< 0.001^×^
Magnesium [mmol/L]	0.78 ± 0.04	[0.76–0.80]	0.85 ± 0.12	[0.77–0.94]	0.87 ± 0.05**††**	[0.83–0.90]	0.039^×^
Blood glucose [mg/dL]	99.75 ± 10.21	[92.68–106.82]	122.00 ± 17.55^∗∗^	[109.84–134.16]	99.25 ± 6.14	[95.00–103.50]	0.014^×^
**Tissue damage marker**							
Creatine Kinase [U/L]	185.3 ± 138.5	[89.3–281.2]	2572.9 ± 1073.5^∗^	[1829.0–3316.8]	2865.4 ± 904.5**†**	[2238.6–3492.1]	0.002^×^
Lactate Dehydrogenase [U/L]	200.0 ± 24.54	[182.99–217.01]	373.8 ± 48.70^∗∗^	[340.00–407.50]	312.2 ± 74.79**†**	[260.34–364.00]	< 0.001^×^
Creatinine [mg/dL]	0.90 ± 0.05	[0.86–0.93]	1.54 ± 0.29^∗∗^	[1.34–1.74]	1.03 ± 0.14	[0.93–1.12]	< 0.001^×^
GFR [mL/min/1.73m^2^]	106.23 ± 7.77	[100.85–111.61]	58.53 ± 14.33^∗∗^	[48.60–68.46]	93.23 ± 13.06	[84.18–102.28]	< 0.001^×^
Blood urea nitrogen [mg/dL]	14.88 ± 4.55	[11.72–18.03]	29.13 ± 6.81^∗∗^	[24.40–33.85]	25.50 ± 4.44**††**	[22.42–28.58]	< 0.001^×^

**FIGURE 4:**
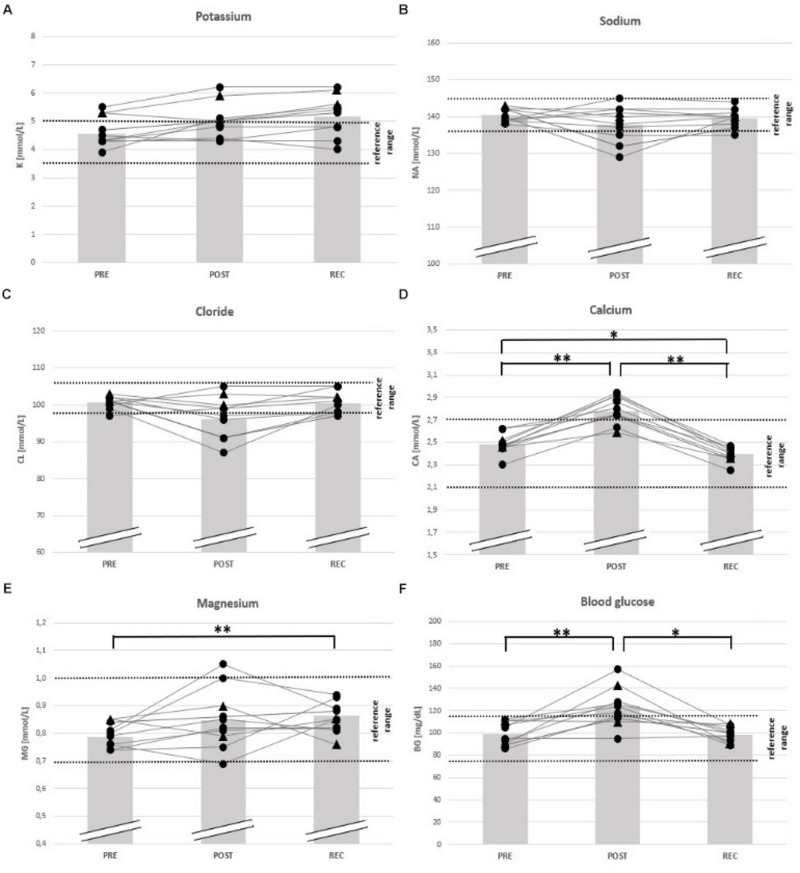
Individual data on blood electrolytes and blood glucose before competition (PRE), immediately after the race (POST) and 24 h after finishing (REC). Changes of potassium **(A)**, sodium **(B)**, chloride **(C)**, calcium **(D)**, magnesium **(E)**, and blood glucose **(F)**. Drop-outs marked triangular. ^∗^*p* < 0.05, ^∗∗^*p* < 0.01.

**FIGURE 5:**
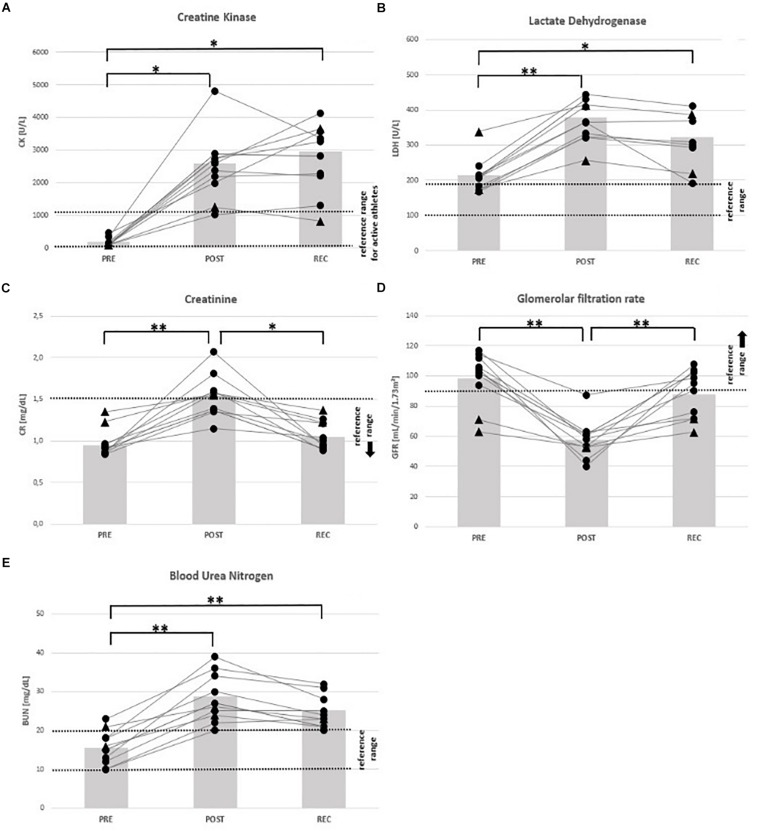
Individual data on markers of muscular damage and renal function before competition (PRE), immediately after the race (POST) and 24 h after finishing (REC). Changes of creatine kinase **(A)**, lactate dehydrogenase **(B)**, creatinine **(C)**, glomerular filtration rate **(D)**, and blood urea nitrogen **(E)**. Drop-outs marked triangular. ^∗^*p* < 0.05, ^∗∗^*p* < 0.01.

*Values are shown as means ± SD and P-values of ANOVAs and post hoc tests. Drop-outs are not included in statistics. See Figures 3–5 for single parameters. GFR, Glomerular filtration rate; p. adjusted p-value of ANOVA; 95% CI, 95% confidence interval. ^×^significance by ANOVA (P < 0.05); ^∗^PRE-POST. P < 0.05; ^∗∗^PRE-POST. P < 0.01; ^†^PRE-REC. P < 0.05; ^††^PRE-REC. P < 0.01.*

**TABLE 5:** Minimum, maximum and number of participants out of medical reference PRE, POST, REC of blood cell counts, serum electrolytes, and tissue damage and renal function.

	**Min/Max**			**OOR (*N* = 8)**		
		
	**PRE**	**POST**	**REC**	**PRE**	**POST**	**REC**
**Blood cell count**						
Erythrocytes [10^6^ μL^–1^]	4.60/5.57	4.58/5.71	4.23/5.19	–	–	1 ↓
Leucocytes [10^3^ μL^–1^]	5.85/8.68	13.46/21.01	4.79/10.16	–	8 ↑	1 ↑
Neutrophils [10^3^ μL^–1^]	3.24/5.27	9.42/18.75	2.15/6.54	–	8 ↑	1 ↑
Monocytes [10^3^ μL^–1^]	0.33/0.48	0.86/1.38	0.27/0.76	–	8 ↑	3 ↑
Eosinophils/Basophils [10^3^ μL^–1^]	0.05/0.39	0.02/0.22	0.08/0.54	n/s	n/s	n/s
Lymphocytes [10^3^ μL^–1^]	1.37/2.80	0.68/2.64	1.34/2.90	–	1 ↓	–
Platelets [10^3^ μL^–1^]	177/305	206/416	186/319	–	1 ↑	–
Hematocrit [%]	39/45	38/45	36/43	2 ↓	1 ↓	5 ↓
**Blood electrolytes, blood glucose**						
Sodium [mEq⋅L^–1^]	138/142	129/145	135/144	–	3 ↓	1 ↓
Chloride [mEq⋅L^–1^]	97/102	87/105	97/105	1 ↓	5 ↓	1 ↓
Potassium [mEq⋅L^–1^]	3.90/5.50	4.30/6.20	4.00/6.20	1 ↑	3 ↑	4 ↑
Calcium [mEq⋅L^–1^]	2.30/2.62	2.63/2.94	2.25/2.47	–	7 ↑	–
Magnesium [mEq⋅L^–1^]	0.74/0.84	0.69/1.05	0.81/0.94	–	1 ↑	–
Blood glucose [mg/dL]	86/112	95/157	89/107	–	3 ↑	–
**Muscular damage, renal function**						
Creatine Kinase [U/L]	74/459	1022/4810	1300/4139	–	7 ↑	8 ↑
Lactate Dehydrogenase [U/L]	167/240	321/444	192/411	5 ↑	8 ↑	8 ↑
Creatinine [mg/dL]	0.84/0.97	1.15/2.07	0.89/1.26	–	4 ↑	–
GFR [mL/min/1.73m^2^]	93.89/116.92	40.0/87.34	71.87/107.7	–	8 ↓	2 ↓
Blood urea nitrogen [mg/dL]	10.0/23.0	20.0/39.0	20.0/32.0	1 ↑	7 ↑	7 ↑

*Values are shown as minimum (Min), maximum (Max). Drop-outs not shown. OOR, out of reference range; GFR, glomerular filtration rate. ↑, above upper range limit; ↓, below lower range limit. See Figures 3–5 for single parameters.*

## Results

### Characteristics and Race Performance

The race was finished between 10.4 and 16.1 h (Table 3) reflecting large heterogeneity with regard to training and race performance. The comparison of age and race time data between the finishers of our study sample and all race participants indicate that study participants represent the whole range of all participants (Table 3). During the competition (3 am to 9 pm), the average ambient air temperature was 20.1°C (17°C to 37°C) and relative humidity was 54% (45% to 61%), measured in the start/finish area 424 m above sea level.

Body mass did not change significantly during the race (average weight loss: − 0.8 kg) but was slightly higher POST in 2 participants (+ 0.7 and + 0.2 kg). Race time was correlated with athletes BMI (*r* = 0.862), but age, training volume and body mass change during the race was not associated with running performance.

### Blood Cell Counts and Electrolytes

From PRE to POST, mean counts of total white blood cells (WBC) (+ 137%), neutrophils (NEU) (+ 215%), monocytes (MONO) (+ 180%) and platelets (PLT) (+ 18%) had increased and eosinophils/basophils (EO/BASO) (− 69%) had decreased. Red blood cells (RBC), lymphocytes (LYM), and hematocrit (Hct) remained unchanged (Table 4). At REC, only MONO were still increased (+ 41%) compared to PRE (Table 4). At POST, counts of total WBC, NEU, and MONO were OOR in all participants (↑) and in 1 person each regarding to LYM (↓), PLT (↑), and Hct (↓) (Table 5). At REC, Hct was OOR in 5 participants (↓), MONO in 3 (↑), RBC (↓), total WBC (↑), and NEU (↑) in 1 person each (Table 5).

With regard to electrolyte changes, only mean concentrations of Ca had increased at POST (+ 13%), whereas Na, Cl, K, and Mg remained unchanged. At POST, also BG had increased (+ 22%) (Table 4). At REC, Ca levels were reduced (− 4%), but Mg was elevated compared to baseline (+ 11%). Other electrolytes and BG remained unchanged from PRE to REC (Table 4). At POST, Ca was OOR in 7 participants (↑), Cl in 5 (↓), and Na in 3 (↓), K in 3 (↑) and Mg in 1 (↑). One day after finishing, one runner was still OOR with regard to Na and Cl (↓), and 4 in K (↑). 3 participants were hyperglycemic in POST, but nobody in REC (Table 5).

### Markers for Muscle Damage and Renal Dysfunction

All mean values of markers for skeletal muscle damage (CK, LDH) and renal dysfunction (CR, BUN, and GFR) had changed at POST: CK (+ 1289%), LDH (+ 87%), CR (+ 72%), BUN (+ 96%), and GFR (− 45%). At REC compared to PRE, CK (+ 1447%), LDH (+ 56%) and BUN (+ 71%) remained high (Table 4).

At POST, all participants were OOR with regard to LDH (↑) and GFR (↓), 7 regarding CK (↑) considering the specific reference for active athletes (< 1083 U/L), and BUN (↑), respectively, and 4 regarding CR (↑). According to the classification of the Acute Kidney Injury Network (Mehta et al., 2007), 5 participants (63%) suffered from AKI stage I, and 2 from stage II (25%). At REC, all participants were OOR regarding CK and LDH (↑), and 7 regarding BUN (↑) (Table 5), 2 were still suffering AKI stage 1. See Figures 3–5 for individual data of every parameter.

## Discussion

The main findings of the present study suggest impaired kidney function in most of the non-elite ultramarathon runners immediately post-race, which persisted in some of them after 24 h of recovery.

The negative relationship between BMI and race performance in ultramarathon runners is in accordance with other studies (Hoffman, 2008). Interestingly, in the population of this study, race performance was correlated neither with age nor training volume, which would be expected to be the case in more elite runners (Knechtle and Nikolaidis, 2018). This may be explained by the fact that study participants were non-elite runners with large interindividual performance differences.

As expected, major increases in indirect markers for potential muscle damage (CK and LDH) have been demonstrated POST and REC in the present study. Ultramarathon running is known to induce muscle tissue damage followed by impaired muscle function and release of CK and LDH into the blood (Brancaccio et al., 2006; Baird et al., 2012; Kim et al., 2016; Knechtle and Nikolaidis, 2018). The severity of muscle damage may be directly linked to the distance covered and seems to be most pronounced after mountain marathons with long downhill-passages (eccentric load) (Brancaccio et al., 2007; Jastrzębski et al., 2015; Kim et al., 2016; Shin et al., 2016; Knechtle and Nikolaidis, 2018). Whereas previous studies reported muscle damage to be associated with age, fitness level and competition experience (Noakes and Carter, 1982; Jastrzębski et al., 2016), we found no associations. This may be explained by the small sample size and/or the heterogeneity of our runners. On average, the presented CK and LDH values are similar to previous findings reported for running distances ranging from marathons to and ultramarathons up to 100 km (Kim et al., 2007; Jastrzębski et al., 2015; Shin et al., 2016).

Although CK may be considered as an indirect marker for muscle damage in athletes, a large variability of CK concentrations post-exercise has been reported (Brancaccio et al., 2007; Kim and Lee, 2015). This may be explained by the dependence of serum CK increase on body composition (muscle mass), metabolic dysfunctions (depletion of nutrients during excessive exercise), inflammatory effects due to immune responses to exercise, electrolyte and hydration disturbances (hypocalcemia, hyper-/hyponatremia, hyper/hypokalemia) which is greatly influenced by ambient temperature (thermal stress) (Brancaccio et al., 2006; Baird et al., 2012; Kim and Lee, 2015; Kim et al., 2016). Moreover, the heterogeneity of serum enzyme levels depends on genetic disposition (low- vs. high-responder) (Brancaccio et al., 2007). Although the CK level in serum is an indirect marker for clinically relevant muscle injury, regular participation in high-volume exercise consistently results in increased serum CK levels, suggesting that CK leakage to the blood is a physiological response to prolonged exercise (Mougios, 2007; Baird et al., 2012). Usually, even distinct elevations of serum enzymes after prolonged and intense exercise do not indicate serious muscle damage because CK and LDH levels regularly return to baseline within some days without medical treatment (Baird et al., 2012). Thus, increased serum CK alone is not an accurate reflection of structural muscle damage and may be a consequence of normal muscular activity (Baird et al., 2012). Rhabdomyolysis, a serious syndrome of muscle injury, is usually associated with CK levels between 10,000–200,000 U/L (Huerta-Alardin et al., 2005; Baird et al., 2012; Kim and Lee, 2015). Although we did not determine additional indicators of potential rhabdomyolysis, such as myoglobin, CK concentrations of our participants do not indicate serious damage of muscle tissue.

Two effects are known to cause leucocytosis during workload: a demargination of WBC into the blood stream (1) and inflammation following tissue injury, where the increase is characterized primarily by NEU and MONO, but not by LYM (2) (Wells et al., 1982; Nieman et al., 1989; Scharhag et al., 2002; Suzuki et al., 2003). Even small exercise-related muscle damage contributes to local and systemic inflammation, depending on age, exercise volume and intensity, and eccentric contribution (Gabriel and Kindermann, 1997; Scharhag et al., 2002; Peake et al., 2005). WBC concentrations measured after various ultramarathons be by are to be 9.4 ^∗^ 10^3^ μL^–1^ after 100 km non-stop running (Jastrzębski et al., 2016) or 13.3 ^∗^ 10^3^ μL^–1^ after 24 h non-stop running with a mean covered distance of 140.3 km (Passaglia et al., 2013). WBC values in the present study are even higher despite shorter race distance and times. Thus, the linearity between stress on the immune system and skeletal muscle damage suggest that our runners suffer from higher muscle damage than similar studies, presumably caused by running downhill passages and heat (Knechtle and Nikolaidis, 2018). However, according to our study, levels of WBC and NEU commonly decrease within 24–48 h after ultramarathon, indicating a normal physiologic process after intensive workload rather than serious damage (Nieman et al., 1989; Scharhag et al., 2002). Increased MONO at REC may indicate inflammation (Nieman et al., 1989).

Kidney dysfunction and AKI are common after excessive exercise indicated by serum CR elevation and eGFR decrease (Hou et al., 2015; Hodgson et al., 2017). This is a consequence of two processes, (1) the physiologic redistribution of blood flow from the kidney to skeletal muscle during exercise and/or (2) the direct toxic effect of released potassium and myoglobin following severe muscular damage (Baird et al., 2012; Hodgson et al., 2017). In ultramarathon studies, about 30–85% of the participants met AKI criteria immediately after competition where most of them were AKI stage 1, which is usually not considered to be of clinical relevance (Hou et al., 2015; Hodgson et al., 2017). Renal dysfunction and serum CR were shown mostly to return to baseline within 24–48 h (Hodgson et al., 2017).

A relatively large percentage of participants in the present study suffered AKI (25% AKI stage II), likely due to only moderate competition experience, thermal stress and the course containing a large part of downhill running (eccentric exercise). Exercise-associated hyponatremia EAH (3 OOR POST) seems not to be an important risk factor in the present study. However, the fact that 25% of our participants still showed AKI stage I at REC and BUN being OOR in 7 runners (88%) at REC may indicate sustained renal dysfunction. Usually, runners with mild AKI do not require medical attention locally, but a review of case reports summarizes that the majority of runners with AKI post-race (21 of 27, 77%) requested medical attention during the consequent days (Hodgson et al., 2017).

Main limitations to be mentioned are the small sample size, the inclusion of only male participants and the lack of monitoring food and fluid intake exactly during the competition and the 24 h of recovery. However, the broad range of blood parameters determined in this heterogenous sample of mainly recreational runners and the fact that REC has been rarely investigated in ultramarathon running, may be considered as strength.

In conclusion, changes in CK and LDH levels and leucocytosis may likely represent relatively harmless “side-effects” of prolonged running in individuals with only moderate ultramarathon experience and training status. However, AKI may become clinically relevant in this population under the given conditions, which should be considered by responsible race managers and medical advisors. Coaches and athletes must be aware of the kidney injury risk related to ultramarathon running and predisposing risk factors such as individual training status and competition experience, age, running distance, route profile, and ambient temperature. Gradual adaptation to such conditions and medical surveillance are most important preventive measures. Future research should focus on the impairment and recovery of post-race kidney function over prolonged periods, i.e., days to weeks, and associated individual risk factors.

## Data Availability Statement

The datasets generated for this study are available on request to the corresponding author.

## Ethics Statement

The studies involving human participants were reviewed and approved by the Review Board Department of Sports Science, University of Innsbruck. The patients/participants provided their written informed consent to participate in this study.

## Author Contributions

FH, MB, EC, EG, and WK-R planned the project. FH, EC, and DP acquired the data. FH, EC, DP, and MB drafted the manuscript. EG, MB, WK-R, and EC supervised the project and provided the resources. All authors contributed to manuscript revision, read, and approved the submitted version.

## Conflict of Interest

FH was temporarily employed during the project by the company Oroboros Instruments, Innsbruck, Austria. EG is the founder and CEO of Oroboros Instruments. The remaining authors declare that the research was conducted in the absence of any commercial or financial relationships that could be construed as a potential conflict of interest.

## Abbreviations

BG: blood glucose
BUN: blood urea nitrogen
Ca: calcium
CK: creatine kinase
CL: chloride
CR: creatinine
GFR: glomerular filtration rate
Hct: haematocrit
K: potassium
LDH: lactate dehydrogenase
LYM: lymphocytes
MG: magnesium
MONO: monocytes
NA: sodium
NEU: neutrophils
PLT: platelets
RBC: red blood cells
WBC: white blood cells.
